# Altered protein dynamics of disease-associated lamin A mutants

**DOI:** 10.1186/1471-2121-5-46

**Published:** 2004-12-13

**Authors:** Susan Gilchrist, Nick Gilbert, Paul Perry, Cecilia Östlund, Howard J Worman, Wendy A Bickmore

**Affiliations:** 1 MRC Human Genetics Unit, Crewe Road, Edinburgh EH4 2XU, UK; 2 Departments of Medicine and of Anatomy and Cell Biology, College of Physicians, Columbia University, New York, NY 10032, USA

## Abstract

**Background:**

Recent interest in the function of the nuclear lamina has been provoked by the discovery of lamin A/C mutations in the laminopathy diseases. However, it is not understood why mutations in lamin A give such a range of tissue-specific phenotypes. Part of the problem in rationalising genotype-phenotype correlations in the laminopathies is our lack of understanding of the function of normal and mutant lamin A. To investigate this we have used photobleaching in human cells to analyse the dynamics of wild-type and mutant lamin A protein at the nuclear periphery.

**Results:**

We have found that a large proportion of wild-type lamin A at the nuclear periphery is immobile, but that there is some slow movement of lamin A within the nuclear lamina. The mobility of an R482W mutant lamin A was indistinguishable from wild-type, but increased mobility of L85R and L530P mutant proteins within the nuclear lamina was found. However, the N195K mutant shows the most enhanced protein mobility, both within the nucleoplasm and within the lamina.

**Conclusion:**

The slow kinetics of lamin A movement is compatible with its incorporation into a stable polymer that only exchanges subunits very slowly. All of the myopathy-associated lamin A mutants that we have studied show increased protein movement compared with wild-type. In contrast, the dynamic behaviour of the lipodystrophy-associated lamin A mutant was indistinguishable from wild-type. This supports the hypothesis that the underlying defect in lamin A function is quite distinct in the laminopathies that affect striated muscle, compared to the diseases that affect adipose tissue. Our data are consistent with an alteration in the stability of the lamin A molecules within the higher-order polymer at the nuclear lamina in myopathies.

## Background

**Figure 1:**
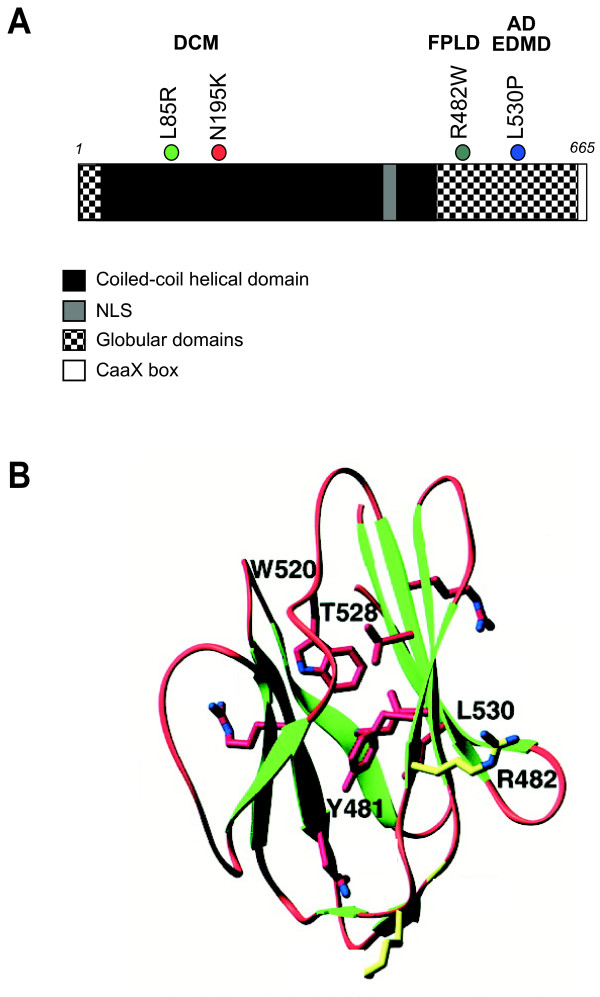
**Structure of lamin A protein.**A) Diagram of lamin A amino acid sequence showing the domains of the protein, and the position of the four laminopathy-associated missense mutations in DCM, FPLD and AD-EDMD. B) Structure of the C-terminal globular domain of Lamin A showing the relative positions of the FPLD associated R482W missense mutation and the AD-EDMD associated L530P mutation. (Adapted with permission from [25]).

The nuclear lamina is a filamentous network of lamin proteins that underlies the inner nuclear membrane (INM). It is thought to make connections between both integral membrane proteins of the INM, and chromatin. It may therefore play a fundamental role in the functional organisation of the nucleus. Lamins are type V intermediate filament (IF) proteins, consisting of a central coiled-coil region, and globular N-terminal and C-terminal domains. The N-terminal domain has a nuclear localisation signal (NLS) and most lamins, except for lamin C, are farnesylated at their carboxy termini via a CaaX motif [1] (Figure 1A). The mammalian genome contains two *lamin B *genes (*LamB1 and 2*) and *lamins A/C*. The latter is alternatively spliced to produce lamins A and C, as well as other minor species. Lamin B is expressed in all cell types and is essential for cell viability. A-type lamins are expressed in more differentiated cells [2] and are non-essential for cell viability [3].

Lamins readily form parallel coiled-coil dimers, which then associate into larger polymers. However, whereas cytoplasmic IF proteins assemble *in vitro *into 10 nm filaments that resemble those formed *in vivo*, lamins assemble *in vitro *into paracrystalline arrays rather than filaments [4]. This suggests that, *in vivo*, assembly of correct lamin higher-order structures requires the interaction with other molecules/proteins. Lamin A certainly has the ability to interact with other proteins, and also to influence their localisation. In the absence of lamin A, emerin relocates from the INM to the endoplasmic reticulum [3,5,6]. The interaction domain with emerin is in the C-terminal domain of lamin A [7,8]. The coiled-coil region can interact with chromatin [9,10] (Figure 1A). There is also an interaction between lamins A/C and the INM proteins LAP2β [11] and muscle-specific nesprin1 [12].

In addition to its localisation at the nuclear lamina, lamin A is also found within the nucleoplasm where it might interact with other nuclear proteins. Interaction and/or co-localisation between lamin A and; Rb, mRNA splicing factors, LAP2, sites of early DNA replication, and specific transcription factors have been reported [13-18].

Recent interest in the function of the nuclear lamina has been provoked by the discovery of lamin A/C mutations in several human diseases, termed the laminopathies [reviewed in [19]]. What is striking about these diseases is that so many apparently disparate phenotypes arise from mutations in one widely expressed gene. The overt phenotypes of the laminopathies can be grouped according to the major cell types that are affected. Striated (skeletal and cardiac) muscle is affected in autosomal dominant Emery-Dreifuss muscular dystrophy (AD-EDMD), limb girdle muscular dystrophy type 1 (LGMD-1B), and dilated cardiomyopathy (DCM). Adipose and bone tissues are affected in familial partial lipodystrophy (FPLD) and mandibuloacral dysplasia (MAD). Charcot-Marie-Tooth neuropathy type 2B1 (CMT2B1) is a demyelination disease of peripheral neurons. Lastly, Hutchinson-Gilford Progeria Syndrome (HGPS) [20,21] and atypical Werner's Syndrome [22] affect multiple tissue types, including many of those involved in the other laminopathies (muscle, fat, bone), and also results in some premature ageing phenotypes. There are currently three main hypotheses for laminopathy disease mechanisms – nuclear weakness, altered nuclear-cytoskeletal interactions, or changes in gene expression [19,23].

To understand the disease pathology of the laminopathies it will be necessary to better characterise the properties of mutant lamin As. The mutations in AD-EDMD are distributed throughout the coiled-coil domain and the first half of the C-terminal globular domain of lamin A. LGMD and DCM appear to be caused mainly by mutations in the coiled-coil domain [19], although an R571S mutation at the end of the globular domain, that affects only lamin C, has been found in a mild case of DCM. [24].

In contrast, FPLD and MAD mutations cluster tightly within part of the C-terminal globular domain. An explanation for this came from structural analysis of this domain. The residues mutated in FPLD and MAD are on the surface (solvent exposed), whereas residues mutated in other laminopathies are located internally within the hydrophobic core of the domain structure [25,26] (Figure. 1B). The latter mutations may therefore have more profound affects on the structure of the mutant protein, whereas FPLD and MAD mutations may leave the overall structure of the lamin A molecule largely unperturbed but might, for example, interfere with protein-protein interactions.

To better understand the affects of laminopathy-associated mutations on lamin A function we have used fluorescence recovery after photobleaching (FRAP) and fluorescence loss in photobleaching (FLIP) to investigate the protein dynamics of GFP-tagged wild-type and disease-associated mutant lamin As in living cells.

## Results and discussion

### Expression of mutant lamin A in human cells

To investigate the biological affect of different mutations on lamin A nuclear localisation and dynamics we expressed epitope tagged forms of the protein, carrying disease-associated missense mutations, in human HT1080 cells. The mutations chosen were; L85R (DCM), N195K (DCM), R482W (FPLD), and L530P (AD-EDMD). Although L85R and N195K are both located within the coiled-coil domain of lamin A, and associated with DCM (Figure 1A), they have been shown to have different behaviours when transiently expressed [27,28]. R482W and L530P are associated with different disease phenotypes (lipodystrophy and myopathy, respectively), and although they are both within the globular domain, R482W is a surface residue, whilst L530P is internal (Figure 1B).

**Figure 2:**
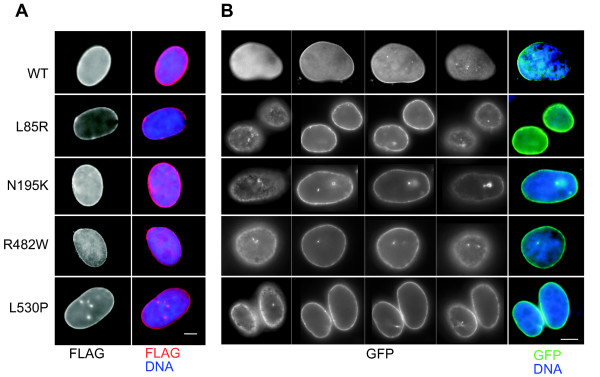
**Sub-cellular localisation of epitope-tagged lamin As.**A) Detection of FLAG-tagged wt and mutant lamin As transfected into human HT1080 fibrosarcoma cells. The FLAG tag was detected by immunofluorescence with M2 anti-FLAG (red in merge), in DAPI stained nuclei (blue in merge). Bar = 10 μm. B) Detection of GFP-tagged wt and mutant lamin As transfected into human HT1080 fibrosarcoma cells. GFP signal in images collected at 2 μm intervals from the top to the bottom of the nucleus is shown in black and white. The merged colour images (far right) show mid-plane images of the GFP signal (green) in DAPI stained nuclei (blue). Bar = 10 μm.

Since these mutations are responsible for autosomal dominant forms of disease they should still exert their molecular phenotype in the cell in the presence of wild-type (wt) protein. Both FLAG-tagged and GFP-tagged prelamin As were transiently transfected into human fibrosarcoma cells. Each protein was processed into mature lamin A [29] and incorporated into the nuclear lamina, as evident by the bright nuclear ring of staining visualised either by immunofluorescence with anti-FLAG antibody or from the GFP signal (Figure 2). The mutant forms of lamin A generally had a more uneven distribution at the nuclear periphery, compared to wt, as has been reported previously [30]. We saw high levels of N195K lamin A in the nucleoplasm in addition to the nuclear periphery, but we did not see much evidence for its aggregation into intra-nuclear foci, as has been reported in mouse myoblasts and embryonic fibroblasts [27,28],. This might reflect differences in cell-type or relative expression levels of the mutant protein. Apparently internal sites of epitope-tagged lamin As are seen, but analysis of 3D image stacks (Figure 2B) shows that these are invaginations of the nuclear periphery and not intra-nuclear foci. Such invaginations has previously been reported in many types of cultured cells [31-33].

### Analysis of lamin A dynamics by FRAP

The lamin A mutations that we have studied are within different domains of the protein (Figure 1A), or within different parts of the same structural domain (Figure 1B). Therefore they likely have different interactions, either with other molecules of lamin A, or with other proteins of the nuclear periphery or nucleoplasm. Such interactions affect the kinetic properties of a protein, and photobleaching and time-lapse imaging can probe this [34]. We therefore analysed the mobility of GFP tagged wt and mutant lamin As by FRAP in transiently transfected human cells.

In each case a region at the nuclear lamina was bleached. The fluorescence within a 1.8 × 1.8 μm region of interest (ROI) of this bleach region was then followed every 5 minutes over a period of up to 65 minutes. To calculate the loss of fluorescence attributed to the imaging process alone, the sum of pixel intensities was also calculated for a control (unbleached) cell in each case. This was used to normalise the fluorescence intensity for each ROI [35]. The mean relative fluorescence intensity for each time point was then calculated for 9 cells of each of the GFP-lamin A proteins (WT, L85R, N195K, R482W and L530P).

**Figure 3:**
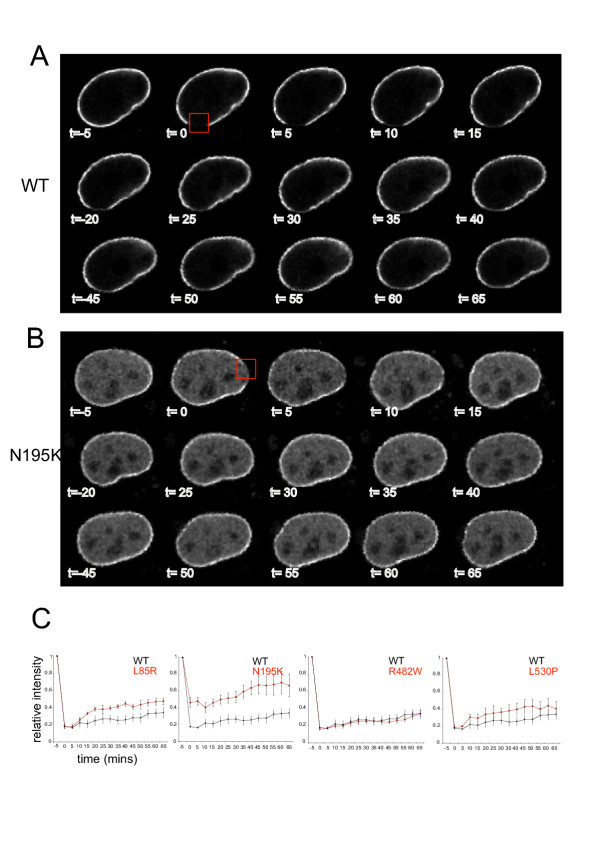
**FRAP analysis of wild type and mutant lamin As. **A and B) Single *z*-plane confocal images of GFP-tagged (A) wt and (B) N195K lamin A expressing cells. Images were captured before (t = -5) and immediately after (t = 0) photobleaching of an area of the nuclear periphery, and at 5 min intervals thereafter. The bleach region is boxed in red. C) Graphs of mean (± s.e.m) relative fluorescence in the bleach area during FRAP, averaged over 9 cells each. In each graph, data for wt (black) and a mutant (red) lamin A are compared.

For wild-type lamin A, fluorescence at the nuclear lamina is visibly bleached (t = 0 in Figure 3A), and only about 20% of the signal recovers over the time course of the experiment (Figure 3C). This indicates that a large proportion (~80%) of lamin A at the nuclear periphery is immobile, at least within the time-frame of these experiments. This is similar to the reported immobility of 60% of lamin B receptor (LBR) in the INM [36]. The recovery curve shows that wt lamin A moves back into the bleach area only very slowly (Figure 3C). The extrapolated t1/2 is ~140 minutes, similar to that reported for lamin B1 (>180 mins) [37]. GFP-tagged lamin C expressed in CHO cells has also been reported to show very little recovery after 1 hour [33]. Most nuclear proteins e.g. transcription factors, and even chromatin-associated proteins such as HP1, are very dynamic with t1/2 values in the range of a few seconds [38]. Even the INM proteins emerin, Lap2β, and Man1 have recovery halftimes of about 1 minute [39]. The slow recovery of lamin A is compatible with its incorporation into a stable polymer that only exchanges subunits very slowly.

The recovery kinetics for the R482W lamin A mutant are indistinguishable from wt and the extrapolated t1/2 = 145 mins (Figure 3C). However, the other lamin A mutants analysed show significant differences. The L85R and L530P mutant proteins appear to be more mobile than wild-type lamin A. They recover more rapidly: t1/2 L85R = 75 mins, L530P = 80 mins. Compared to wt, a higher proportion of the L85R fluorescence (35%) also recovers, suggesting that less of this mutant lamin A is in an immobile fraction.

The most dramatic difference in dynamics was seen for the N195K mutant. Compared to the other lamin As it does not bleach to the same extent, and this is attributable to rapid diffusion of the high levels of nucleoplasmic protein, since at t = 0 recovery of fluorescence can be seen in the nucleoplasmic part of the bleach region, but not in the nuclear periphery itself (Figure 3B). It is known that in early G1 cells the nucleoplasmic pools of lamin A recovery their fluorescence immediately following bleaching [37]. However, even within the nuclear periphery fluorescence recovers within the observation period (Figure 3B, t = 15) and the t1/2 = 30 mins (Figure 3C). Therefore the N195K lamin A mutant is considerably more mobile within the nuclear lamina than wt lamin A, or indeed the other lamin A mutants studied here.

### Analysis of lamin A dynamics by FLIP

**Figure 4:**
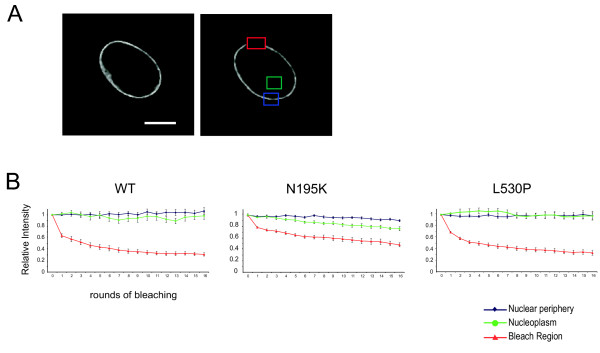
**FLIP analysis of wild type and mutant lamin As. **A) Single *z*-plane confocal images of a GFP-tagged wt lamin A expressing cell captured before (left) and immediately after (right) a round of photobleaching of an area of the nuclear periphery (red box). Fluorescence was also recorded for an unbleached area (blue box) of the nuclear periphery, and a region of the nucleoplasm (green box). Bar = 10 μm **B**) Graphs of mean (± s.e.m) relative fluorescence in the bleach area (red) during successive rounds of FLIP, and in unbleached regions of the nuclear periphery (blue). and the nucleoplasm (green). Data are averaged over 10 cells each for wt lamin A and for the L530P and N195K mutant lamin As.

To further analyse the movement of lamin A within the nuclear lamina, and between the lamina and the nucleoplasm, FLIP experiments were performed on wt, and N195K and L530P mutant GFP-lamin A expressing cells. After successive rounds of photobleaching at a region of the nuclear periphery, the fluorescence at a region of the nuclear periphery distant from the bleach, and at a region within the nucleoplasm were measured (Figure 4) in 10 cells each. As in FRAP, the data was normalised for the loss of fluorescence caused by the successive rounds of imaging.

For both wt and L530P lamin A there is little loss of fluorescence from either a distant region of the nuclear periphery, or the nucleoplasm after repeated rounds of photobleaching (Figure 4B). This reflects the slow FRAP recovery kinetics of these forms of lamin A (Figure 3). In contrast, the nucleoplasmic fraction of the N195K mutant lamin A shows a substantial decrease (24%) in fluorescence after successive rounds of bleaching at the nuclear periphery. This may reflect diffusion into the small region of nucleoplasm contained within the bleach region, but could also be due to exchange of protein between the nucleoplasm and the lamina. A 10% decrease in fluorescence is also seen at a non-bleached part of the nuclear periphery. This suggests that there is enhanced lateral movement of mutant lamin A within the nuclear lamina compared to wild-type protein.

## Conclusions

For GFP-tagged wild-type lamin A we have determined that a large proportion of the protein at the nuclear periphery is immobile (Figure 3), and that any recovery of fluorescence that does occur there is very slow (t1/2 = ~140 mins). This is consistent with the slow recovery halftimes of lamin B1 [37], and the incorporation of lamin A into a stable IF polymer at the nuclear lamina.

Of the four laminopathy-associated mutant forms of lamin A studied by photobleaching all, except for R482, show altered dynamics relative to wt protein. The R482W mutation is associated with FPLD, and other lamin A mutations found in this disease are also either a loss of positive charge at R482, or K486, or the gain of a negative charge (G465D). The amino acid residues involved all map to a solvent-exposed surface in the structure of the Ig-like C-terminal domain [26] (Figure 1B). By NMR and circular dichroism the structure and thermostability of the R482W mutant is similar to that of wt lamin A [26]. Our analysis suggests that the dynamics of the R482W mutant protein within the cell are also similar to wt. It has been suggested that FPLD-associated mutations of lamin A do not destabilise the Ig-like domain of lamin A, but may alter the interaction of the protein with other cellular components. The Ig-like lamin A domain interacts with LAP2α [16], emerin [28], DNA [10] and SREBP1 [18]. Emerin can still interact with R482W lamin A [16], though altered emerin-lamin A interactions have been reported for the R482L mutation [40]. Mutations at R482 have a 5-fold lower affinity for DNA binding in *in vitro *assays [10], and a slightly lower affinity for SREBP1 [18]. We suggest that if lamin A-protein or -DNA interactions are perturbed by the R482W mutation they are not sufficient to affect the dynamics of lamin A movement within the nucleus.

The EDMD-associated L530P mutation is also within the Ig-like domain (Figure 1), but unlike R482W it is located inside of the structure and so is predicted to destabilise protein folding [25,26]. Compared with wild-type and R482W lamin A, we detected increased mobility of L530P lamin A within the nuclear lamina by FRAP (Figure 3). Expression of L530P has been reported to result in decreased emerin localisation at the INM [30]. Therefore, the stability of both emerin and lamin A at the nuclear periphery may be mutually dependent. In the absence of lamin A, emerin completely fails to localise at the INM [3,5,6]. Our analysis of protein dynamics suggests that an altered interaction between emerin and lamin A could alter the stability of the nuclear lamina, reflected in the increased mobility of lamin A.

Missense mutations in the coiled-coil domain of lamin A are associated with the myopathies, not FPLD (Figure 1). They likely impair the dimerization and formation of higher-order filaments of lamin A. The increased mobility of the L85R mutant lamin A, as assayed by FRAP (Figure 3), would be consistent with this. The most dramatic change in lamin A dynamics was seen with the N195K form. FRAP indicates that it is considerably more mobile than wt lamin A (Figure 3). FLIP suggests that there might be exchange between the nucleoplasmic and lamina pools of this mutant protein, as well as enhanced mobility within the nuclear lamina (Figure 4). Like L530P, this mutation is also in the coiled-coil domain, but clearly has a more drastic affect on lamin polymerisation and intra-nuclear dynamics.

Given the genetically dominant nature of many of the laminopathies, it would be interesting to determine whether the presence of a mutant lamin A has an affect on the mobility of the (GFP-tagged) wild-type protein in the same cells.

FLPD is clinically distinct from AD-EDMD and DCM. Patients with FPLD do not have striated muscle pathology, conversely adipose tissue is normal in AD-EDMD and DCM. Whereas we find increased mobility of all the myopathy-associated lamin A mutants we studied, we cannot distinguish between the protein dynamics of wt and an FLPD mutant form of lamin A (R482W). We conclude that in AD-EDMD and DCM laminopathies the structure of the nuclear lamina is perturbed in such a way as to allow for more rapid exchange of lamin A molecules. In contrast, we suggest that, in this respect, the structure of the lamin polymer is normal in FPLD.

## Methods

### Generation of GFP-tagged lamin A constructs

Green Fluorescent Protein (GFP)-tagged human lamin A cloned in pCDNA-3-EGFP (GFP-HLA) was obtained from L Karnitz (Mayo Clinic, Rochester). GFP-tagged mutant lamin As were then generated by transfer from N-terminal FLAG-tagged fusion constructs [27]. GFP-HLA was digested with AccI/EcoRI and fragments of 5.4 Kb (the vector backbone) and 894 bp (GFP coding sequence plus 0–175 bp of lamin A) were purified. FLAG-pre-lamin A coding sequences (carrying laminopathy mutations) were then excised from each of the pSVK vectors using EcoRI/SalI, and cloned into XhoI/EcoRI digested pUC21. A 1.8 kb AccI/SpeI fragment of the coding sequence (removing the FLAG tag and the first 175 bp of lamin A) was purified. The AccI site is upstream of each mutant codon and the SpeI site is downstream of the stop codon. A three way ligation was then performed of this fragment together with the 5.4 Kb and 894 bp AccI/EcoRI fragments from GFP-HLA.

### Cell transfection and immunofluorescence

Human HT1080 fibrosarcoma cells were transfected with plasmids using Lipofectamine™2000 according to the manufacturer's recommendations. Cells grown on glass slides were fixed 24 hours later for immunofluorescence or GFP analysis. Cells were fixed for 10 mins in 4% paraformaldehyde and permeabilised for 10 mins in 0.05% Triton-X100. FLAG-tagged proteins were detected with 1:200 dilution of M2 anti-Flag mouse monoclonal antibody (Sigma) and 1:100 anti-mouse Texas Red Fab'2 heavy and light chains (Jackson Labs). Slides were counterstained with DAPI and analysed using a Zeiss Axioplan microscope fitted with a Xillig CCD camera and a focus motor to collect images at 0.5 μm intervals in the z plane [41].

### Live cell analysis

Cells were grown on DeltaT 0.17 mm culture dishes (Bioptechs Inc) and were mounted onto a heated stage (Bioptechs Inc) on a Zeiss LSM510 confocal microscope. An objective warmer (Bioptechs Inc) was also used to help to maintain a stable temperature of the medium in the culture dish.

### Photobleaching

For FRAP, a 1.8 × 1.8 μm region at the nuclear periphery in the mid-focal plane was bleached with 100 iterations at 100% power of the argon laser running at 6.1 mA (50% power). The pinhole size for the confocal was set at 1 Airy unit. The time series software option was used to specify the appropriate time delay between rounds of 3D image stack capture. Each bleached cell was imaged with a ×100 objective, in a window that included other non-bleached cells to allow for relative fluorescence levels to be normalised. Immediately following the bleach, images in the same z-plane were captured at 1s intervals (t = 0 in Figure 3). Thereafter 3D z-plane stacks were captured of the cell at 5 min intervals for a further 65 mins, using 8% of laser power.

Because of the length of FRAP analysis, nuclear rotation, cell movement and focus drift presented a problem in registering the bleach ROI between time points. To account for this, the best *z*-plane image for the bleach ROI was selected from each time point 3D stack. Each of these was then processed by an interactive rotation script (v3.6 IPLAB, Scanalytics) to correct for nuclear rotation and cell movement. This enabled all images to be superimposed with the pre-bleach image.

For FLIP, an ROI at the nuclear periphery was bleached with 10 laser iterations at 100% of 50% total laser output (~6.1 mA). Following the bleach, 5 images were taken at 2 sec intervals using 8% of laser output. The bleach procedure was repeated for 16 rounds.

In both FRAP and FLIP, the loss of fluorescence attributed to the imaging process alone was assessed from the sum of pixel intensities in a control (unbleached) cell, in each analysis. The relative fluorescence intensity over time was calculated for each defined ROI using a normalisation equation [35].

## Authors' contributions

SG constructed the GFP-tagged lamin A constructs, did the cell transfections, the fluorescence microscopy and the photobleaching analysis. NG gave assistance and advice in the photobleaching studies. PP advised and assisted in confocal microscopy and wrote the scripts for image registration over the time course of FRAP. CO and HJW constructed the FLAG-tagged lamin A mutants and provided advice. WAB conceived of the study and drafted the manuscript. All authors read and approved the final manuscript
